# Modelling the impact of initiation delay, duration and prior PrEP on the efficacy of post‐exposure prophylaxis containing a tenofovir/emtricitabine backbone

**DOI:** 10.1002/jia2.26454

**Published:** 2025-06-26

**Authors:** Lanxin Zhang, Simon Collins, Julie Fox, Max von Kleist

**Affiliations:** ^1^ Project group 5 “Systems Medicine of Infectious Disease”, Robert‐Koch Institute Nordufer 20 Berlin 13353 Germany; ^2^ HIV i‐Base London UK; ^3^ Department of Infectious Disease King's College London London UK; ^4^ Mathematics for Data Science Department of Mathematics and Computer Science Freie Universität Berlin Berlin Germany

**Keywords:** HIV, mathematical modelling, post‐exposure prophylaxis, pre‐exposure prophylaxis, quantitative systems pharmacology, TDF/FTC

## Abstract

**Introduction:**

Pre‐ and post‐exposure prophylaxis (PrEP and PEP) are important pillars of the HIV prevention portfolio to reduce the risk of acquisition just before or after HIV exposure. While PrEP efficacy has been elucidated in many randomized clinical trials, corresponding data for PEP is extremely difficult to obtain in a controlled setting. Consequently, it is almost impossible to study the impact of PEP initiation delay and duration on HIV risk reduction clinically, which would inform recommendations on PEP use.

**Methods:**

We employ pharmacokinetics, pharmacodynamics and viral dynamics models, along with individual factors, such as drug adherence to investigate the impact of initiation delay and PEP duration on HIV risk reduction. We evaluated PEP using two‐ and three‐drug regimens with a TDF/FTC backbone. Moreover, we study PEP efficacy in the context of PrEP‐to‐PEP transitions.

**Results:**

In our simulations, early initiation of PEP emerged as a pivotal factor for HIV risk reduction. We found that 2‐drug (TDF/FTC) PEP may insufficiently protect when initiated > 1 hour post‐exposure. When adding a third drug, early initiation was still a critical factor; however, over 90% efficacy could be achieved when PEP was initiated 48 hours post‐exposure and taken for at least 14–28 days, depending on the efficacy of the third‐drug component. When investigating PrEP‐PEP transitions, we observed that preceding PrEP can (1) contribute directly to prophylactic efficacy, and (2) boost subsequent PEP efficacy by delaying initial viral dynamics and building‐up drug concentrations, overall facilitating self‐managed transitioning between PrEP and PEP.

**Conclusions:**

Our study confirms the critical role of early (< 48 hours) PEP initiation, preferably with three drugs taken for 28 days. Self‐start with TDF/FTC and later addition of a third drug is better than not self‐starting. Furthermore, our study highlights the synergy between recent PrEP intake and PEP and may help to inform recommendations on PEP use.

## 1 INTRODUCTION

An estimated 1.3 million individuals acquired the human immunodeficiency virus (HIV) in 2023 [1]. To date, with a handful of exceptions, there is no cure [2]. However, treatment with antiviral drugs prevents AIDS, as well as transmission [3, 4]. Currently, treatment needs to be taken life‐long, which, in addition to individual burden, requires treatment availability, medical care infrastructure and funding. HIV prevention through vaccination would constitute an ideal means to fight the pandemic. However, all recent vaccine trials prematurely terminated due to failure in demonstrating clinical efficacy [5]. In the absence of effective vaccines, pre‐exposure prophylaxis (PrEP) has partly taken its place. Four effective regimens are currently available: once daily emtricitabine (FTC) with either tenofovir disoproxil fumarate (TDF) or tenofovir alafenamide (TAF) can be administered orally, long‐acting cabotegravir (CAB) can be injected every 2 months. Monthly dapivirine (DPV) vaginal rings to prevent acquisition through receptive vaginal intercourse recently received a positive review by the European Medicines Agency. PrEP with twice‐yearly injectable lenacapavir is submitted for review by regulatory agencies. Of the available PrEP options, oral TDF/FTC is widely available as a generic and rolled out in both low‐ and high‐income countries.

Post‐exposure prophylaxis (PEP) taken after suspected sexual or occupational exposure to HIV [6] denotes another important preventive measure to reduce acquisition risk. Current guidelines recommend to initiate oral PEP within 72 hours after suspected virus exposure and to continue the regimen for 28 days [6, 7, 8]. National [6, 8] and international guidelines [7] differ with regard to recommending two‐ or three‐drug regimens for PEP: For example, TDF/FTC + raltegravir or dolutegravir (DTG) are recommended in the United States, whereas the WHO 2014 guidelines also discuss scenarios where two‐drug regimens with generics may be recommended. To date, TDF/FTC denotes the preferred backbone in PEP, whereas different choices of third‐component drugs may be used [9]. However, because of operational and ethical challenges, no randomized controlled trial has been conducted to test PEP efficacy directly. Current evidence for non‐occupational PEP efficacy has been synthesized from animal transmission models and observational and case studies of PEP use [6, 8]. However, results from observational studies may be impacted by many factors such as individual adherence and risk behaviour [10] and differences in regard to utilized PEP drugs [8]. Although the developed guidelines are based on impressive trans‐disciplinary synthesis of evidence across heterogeneous data sources, it has not been possible to elucidate the sensitivity of a particular PEP regimen to delays in initiation, PEP duration, as well as the impact of PrEP on PEP efficacy.

**Figure 1:**
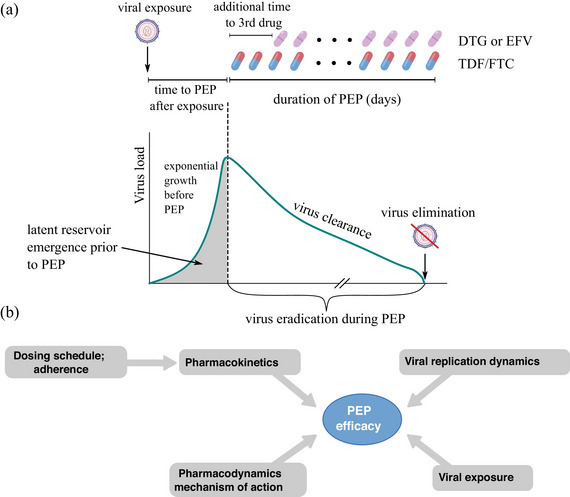
**Schematic of post‐exposure prophylaxis and key parameters influencing PEP efficacy in the mathematical model**. (a) PEP is initiated after virus exposure. In the time between virus exposure and PEP initiation, virus may grow exponentially. The total amount of virus replication during this time may be related to the probability of emergence of latently infected cells, which render infection irreversible (grey area). Depending on the duration of viral growth before PEP and conditioned that latent infected cells have not yet emerged, PEP must be taken long enough to ensure that all replicating viruses are eliminated. (b) Schematic of model constituents for estimating PEP efficacy. Pharmacokinetic models relate arbitrary dosing patterns to target site concentration‐time profiles. Through mechanism‐of‐action models, we predict their impact of early viral dynamics. Lastly, we compute the probability that all virus compartments will be eliminated during PEP, after a particular viral exposure occurred. We use these integrated models to calculate the *per exposure* reduction in HIV acquisition probability (PEP efficacy). Abbreviations: DTG, dolutegravir; EFV, efavirenz; FTC, emtricitabine; PEP, post‐exposure prophylaxis; TDF, tenofovir disoproxil fumarate.

In the absence of randomized controlled trial data on PEP efficacy, mathematical modelling may support the synthesis of evidence, by integrating available knowledge on drug pharmacokinetics (PK), as well as early viral dynamics. However, to our knowledge, no such modelling exists to date. To analyse PEP efficacy for two‐ and three‐drug regimens, to test the impact of delays in “time to PEP,” PEP duration Figure 1a, as well as the transition from PrEP to PEP, we utilized an integrated mathematical model combining drug PK at their target site [11, 12, 13], mechanistic models of direct drug action [14], initial viral dynamics [15] and viral exposure [16] (Figure 1b).

## 2 METHODS

We combined population PK models of oral FTC, TDF, DTG and efavirenz (EFV), an older generation antiretroviral [12, 17, 18, 19], with viral dynamics models [20, 21] and a novel numerical scheme [15] to estimate the prophylactic efficacy of PEP for different dosing patterns, as well as PrEP‐to‐PEP transitions. While individual models had been validated previously [11, 12, 13, 22], the overarching goal of this study was to understand the sensitivity of PEP efficacy towards delay in PEP initiation after virus exposure, as well as the duration of PEP, with and without prior PrEP utilization.

### 2.1 Prophylactic efficacy

In clinical trials, *average* HIV risk reduction is quantified in terms of incidence reduction in an intervention versus a control arm [23, 24, 25, 26]. In a mathematical model of within‐host viral replication, the same quantity can be derived by computing the reduction of infection probability *per viral exposure* due to a prophylactic regimen *S*:

(1) $$\phi =1-\frac{P_{I}\left(Y_{t},S\right)}{P_{I}\left(Y_{t},\emptyset \right)},$$

where *P_I_(Y_t_, S)* and *P_I_* (*Y_t_*, ∅) denote the infection probability in the presence and absence of a prophylactic regimen *S* when *Y_t_* drug‐susceptible viral particles enter a replication‐enabling compartment at time *t*. Notably, the infection probability is the complement of the virus elimination probability *P_E_(Y_t_, S)*.

### 2.2 Virus exposure model

We used previously developed exposure models for sex without condoms [16]. In these models, the number of infectious viruses (inoculum size *Y_t_*) that are transmitted to and reaching an anatomical site where they may spark an infection, are estimated from a binomial distribution, *Y_t_* ∼ *B*(VL*, r*), where VL denotes the donor virus load, and the “success rate” *r* depended on the type of exposure. Throughout this study, unless stated otherwise, we utilize the exposure model designed for receptive vaginal intercourse.

### 2.3 HIV viral dynamics model

To compute the viral elimination probability in the exposed host for prophylactic regimen *S*, we employ a within‐host viral dynamics model [20, 21], depicted in Text S1. The model considers replication of free infectious viruses, early and productively infected T cells, as well as long‐lived cells such as macrophages and latently infected T cells, which are believed to be an obstacle for the within‐host clearance of HIV [27]. The model was derived from first principles [20] and allows to model pharmacodynamic (PD) effects of all antiviral classes [22]. Moreover, it allows to incorporate state‐of‐the‐art population PK models.

### 2.4 Pharmacokinetics

**Figure 2:**
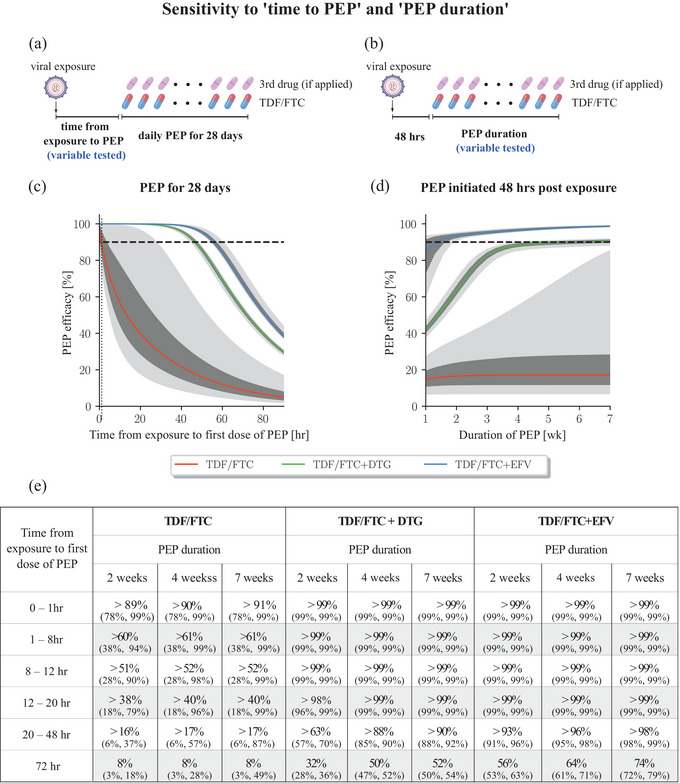
**Sensitivity of TDF/FTC‐based PEP on initiation delay and PEP duration**. (a) Schematic of the dosing regimen in panel C, where the variable tested is “time to PEP.” (b) Schematic of the dosing regimen in panel D, where the variable tested is “PEP duration.” (c) PEP efficacy of TDF/FTC (red line), TDF/FTC + EFV (blue line) or TDF/FTC + DTG (green line) when initiated at different delays post virus exposure and taken for 28 days once‐daily. The vertical dotted line indicates PEP initiation 1 hour after exposure. (d) Efficacy of TDF/FTC (red line), TDF/FTC + EFV (blue line) and TDF/FTC + DTG (green line) when initiated 48 hours post virus exposure and taken for different durations. (e) Numerical results for different “times to PEP,” “PEP durations” and regimen. Values denote the median efficacy and 95% confidence interval evaluated at the maximum “time to PEP” of the indicated interval (e.g. 8 hours for the 2–8 hours interval). All computations were conducted on 1000 virtual patients. The daily oral dose for each drug corresponds to 300/200 mg TDF/FTC, 50 mg DTG and 400 mg EFV. The coloured lines depict the median predicted PEP efficacy, whereas the dark‐ and light grey areas present the inter‐quartile range and the 95% confidence range, respectively. Dashed horizontal lines indicate 90% prophylactic efficacy. Abbreviations: DTG, dolutegravir; EFV, efavirenz; FTC, emtricitabine; PEP, post‐exposure prophylaxis; TDF, tenofovir disoproxil fumarate.

**Figure 3:**
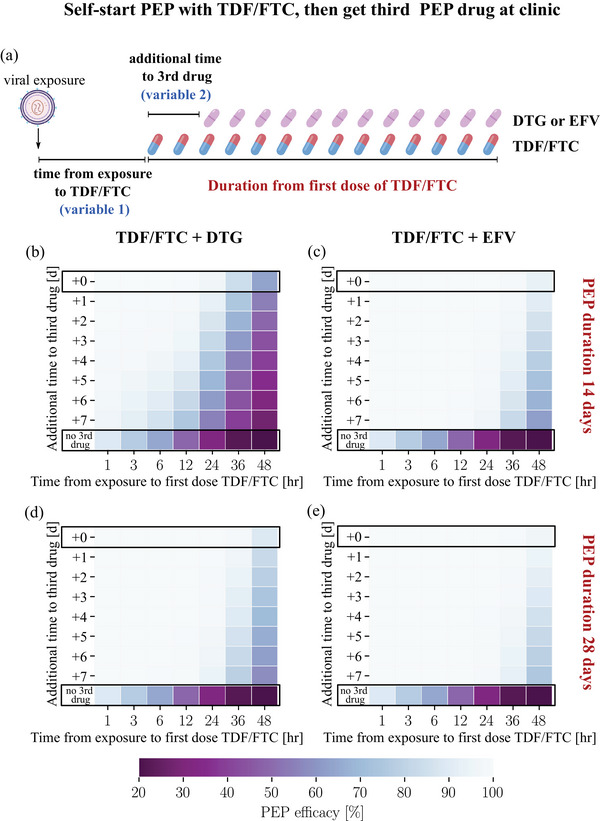
**Efficacy of TDF/FTC‐based PEP with delayed initiation of TDF/FTC and further delay of the third drug**. (a) Schematic of the dosing regimen. For the drug combinations TDF/FTC + DTG and TDF/FTC + EFV, PEP efficacy was computed for virus exposures occurring within 1–48 hours before the first dose of TDF/FTC. The third drug was then added to the PEP regimen 1–7 days after the first dose of TDF/FTC. (b) PEP efficacy for the drug combination TDF/FTC + DTG, PEP duration was 14 days from the first dose of TDF/FTC. (c) Corresponding PEP efficacy for TDF/FTC + EFV. (d) PEP efficacy for TDF/FTC + DTG when taken for 28 days after the first TDF/FTC dose. (e) Corresponding PEP efficacy for TDF/FTC + EFV. The daily oral dose for each drug corresponds to 300/200 mg TDF/FTC, 50 mg DTG and 400 mg EFV. In panels B–E, the top row outlined in black denotes the scenario where the third drug is immediately added to the TDF/FTC backbone; the bottom row represents the scenario where no third drug was added to the TDF/FTC backbone. DTG, dolutegravir; EFV, efavirenz; FTC, emtricitabine; PEP, post‐exposure prophylaxis; TDF, tenofovir disoproxil fumarate.

**Figure 4:**
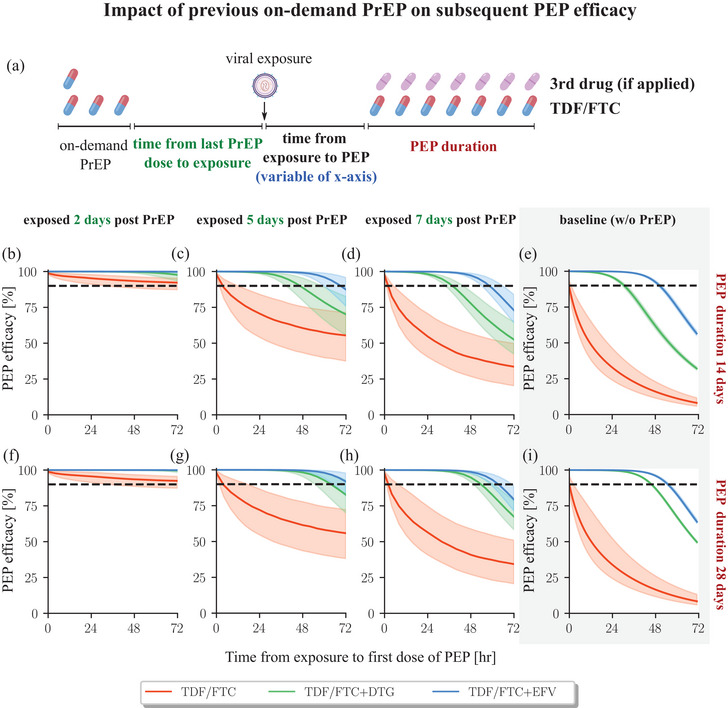
**PEP efficacy following on‐demand PrEP**. (a) Schematic of the dosing regimen. TDF/FTC was initially administered as “on‐demand” PrEP (2‐1‐1), followed by viral exposure after a certain period. Subsequently, the PEP regimen was initiated after various time intervals, potentially incorporating a third drug. (b–d) The efficacy profiles for PEP with overall duration of 14 days, and the exposure occurred 2, 5 and 7 days after the on‐demand PrEP, respectively. (f–h): The efficacy profiles for PEP with overall duration of 28 days. (e and i): PEP efficacy of baseline scenario without preceding PrEP. All computations were performed on 1000 virtual patients. The daily dose for each drug corresponds to 200 mg FTC, 300 mg TDF, 50 mg DTG and 400 mg EFV. The coloured lines represent the median efficacy value in cases where PEP was initiated at the respective time point along the x‐axis. Dashed horizontal lines indicate 90% prophylactic efficacy. The shaded areas depict the quantile range of prophylactic efficacy. Abbreviations: DTG, dolutegravir; EFV, efavirenz; FTC, emtricitabine; PEP, post‐exposure prophylaxis; PrEP, pre‐exposure prophylaxis; TDF, tenofovir disoproxil fumarate.

**Figure 5:**
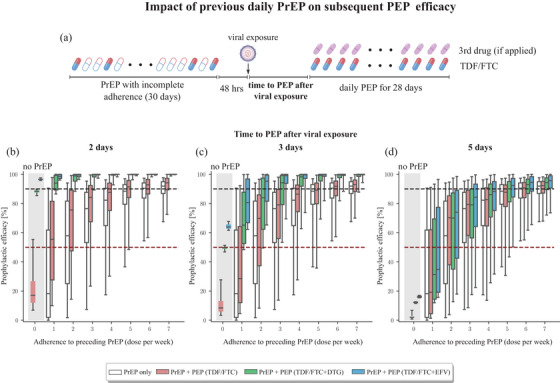
**Predicted efficacy of once‐daily PEP, in cases where PrEP was recently taken**. (a) Schematic of dosing regimen: PrEP with incomplete, variable levels of adherence was taken and stopped 48 hours before virus exposure. PEP with either TDF/FTC, or TDF/FTC + DTG or EFV was then initiated after a variable delay and taken for 28 days. PEP efficacy is calculated with regard to preceding PrEP adherence, as well as delay in PEP initiation. (b–d) Computed prophylactic efficacy for the distinct PrEP+PEP regimen, if PEP was initiated 2, 3 or 5 days post‐exposure and taken daily for 28 days. The daily oral dose for each drug corresponds to 300/200 mg TDF/FTC, 50 mg DTG and 400 mg EFV. The grey‐shaded area indicates PEP efficacy, with no prior PrEP, while empty boxplots highlight the prophylactic effect of preceding PrEP, without subsequent PEP. Boxplots show the median, interquartile ranges and whiskers encompass the 95% confidence interval. Dashed red lines indicate 50% prophylactic efficacy, while dashed black lines indicate 90% prophylactic efficacy. Abbreviations: DTG, dolutegravir; EFV, efavirenz; FTC, emtricitabine; PEP, post‐exposure prophylaxis; PrEP, pre‐exposure prophylaxis; TDF, tenofovir disoproxil fumarate.

We used the previously developed PK models of FTC [18] and TDF [18], which allow to predict prodrug PK in blood plasma, as well as the PK of the active phosphorylated moieties in peripheral blood mononuclear cells (PBMCs). In line with recent findings [11], we assume that the concentration of tenofovir‐diphosphate (TFV‐DP) and emtricitabine‐triphosphate (FTC‐TP) in PBMCs predict prophylactic effect. Furthermore, we adopted PK models for DTG [12] and EFV [13]. To capture the impact of individual PK variability, we sampled PK parameters for 1000 virtual patients per drug, utilizing distributions described in the aforementioned original sources. We considered oral doses of 300/200, 50 and 400 mg for TDF/FTC, DTG and EFV and daily dosing schedules as depicted in Figures 2, 3, 4, 5.

### 2.5 PK‐PD link

To evaluate the combinatorial effect of FTC‐TP and TFV‐DP (the active intracellular components of TDF/FTC), we adopted a model for the molecular mechanism of action and drug–drug interaction [14]. For DTG and EFV, their direct effect can be modelled using the Emax equation [28], corrected by plasma protein binding [12, 13], and was assumed to be additive with the TDF/FTC backbone, due to lack of evidence for non‐additivity (DTG), or a lack of parameters to describe synergy (EFV).

### 2.6 Numerics

We adopted the numerical scheme from [15] to formulate a set of ordinary differential equations that allows computing extinction probabilities *P_E_(Y_t_, S*) of each compartment of the viral dynamics model, subject to PK and PD of the considered drugs, Equation (S16) in Text S1.

### 2.7 Software availability

Computer codes are available at https://github.com/KleistLab/PEP under the MIT license [29].

## 3 RESULTS

### 3.1 “Time to PEP” is the most critical parameter

Currently, the WHO recommends to initiate PEP up to 3 days after potential viral exposure and to continue PEP for 28 days [7]. Using our modelling framework, we evaluated how PEP initiation delay may alter prophylactic efficacy. As a first test case, we explored the efficacy of 2‐drug (oral TDF/FTC) PEP, as these drugs may be available in many settings where PrEP is implemented. We created 1000 virtual individuals and simulated individual PK based on the dosing profiles in Figure 2a. Using the model, we then computed the prophylactic efficacy for each virtual individual, if a 2‐drug PEP with daily TDF/FTC was initiated at different time points post‐exposure with drug‐susceptible virus and taken for 28 days. Figure 2c depicts summary statistics of derived PEP efficacy estimates across the cohort of virtual individuals (median, interquartile ranges and 95% confidence intervals). From the simulations, it is evident that ≥ 90% 2‐drug PEP efficacy is only achieved if TDF/FTC is initiated within 1 hour after virus exposure. Efficacy steeply drops to *<* 50% when TDF/FTC‐PEP was initiated 20 hours after virus exposure. We also found that a longer duration of 2‐drug TDF/FTC PEP could not compensate for delayed initiation (Figure 2b,d red line) with efficacy remaining low (median efficacy *<* 20%), when PEP was initiated 48 hours after virus exposure and taken for up to 7 weeks. We tested whether a third drug component (DTG or EFV) may impact on prophylactic efficacy and change sensitivity to “time to PEP” and “PEP duration,” Figures 2a–d. Compared to 2‐drug PEP, 3‐drug PEP provided *>* 88% protection against sexual transmission, when initiated 48 hours post‐exposure and continued for 4 weeks (Figure 2e). When initiated 48 hours post‐exposure, we predicted that TDF/FTC + EFV provided 91–96% HIV risk reduction when taken at least for 2 weeks, whereas TDF/FTC + DTG provided 85–90% HIV risk reduction when taken for at least 4 weeks. In contrast to 2‐drug PEP, we predicted that PEP efficacy with TDF/FTC + EFV or DTG can increase with an extended duration of PEP.

### 3.2 Third drug may be added later, if TDF/FTC is initiated quickly

In many settings, all three drugs may not be available within a reasonable time. However, TDF/FTC may be readily available to individuals who already used, or have access to PrEP. We investigated whether PEP initiation with TDF/FTC (“self‐start”) and later addition of a third drug may effectively prevent acquisition (schematic in Figure 3a). Reading Figures 3b–e bottom‐to‐top indicates that adding DTG or EFV to a TDF/FTC backbone increases PEP efficacy (lowest row: TDF/FTC only) and that earlier addition of the third drug results in greater efficacy (top row). Reading Figures 3b–e horizontally (left‐to‐right) indicates that the earlier TDF/FTC is initiated, the better. For the three‐drug combinations, a temporal “window of opportunity” arises, where the PEP efficacy exceeds 90%. For TDF/FTC + DTG, the duration of PEP strongly impacts on its prophylactic efficacy (compare panels B and D in Figure 3), whereas the impact is less strong for PEP with TDF/FTC + EFV, which is already efficient when taken for 2 weeks. The simulations highlight that if TDF/FTC is available within 12–24 hours, the third drug should be added within a week, and PEP should preferably be taken for 28 days from the first TDF/TFC dose. Efficacy did not change when TDF/FTC was initiated with a double dose (Figure S2).

### 3.3 Previous PrEP can boost subsequent PEP efficacy and widen the “window of opportunity”

The pharmacologically active components of TDF and FTC (TFV‐DP and FTC‐TP, respectively) are built‐up slowly within HIV target cells [16, 17, 18, 30], necessitating almost instantaneous initiation of 2‐drug (TDF/FTC) PEP (Figure 2c). However, due to the long half‐life of TFV‐DP and FTC‐TP in PBMCs (4–7 and 1–2.2 days, respectively [31, 32, 33, 34, 35], they may persist if PrEP had been taken in the past. To assess the combined impact of past TDF/FTC PrEP intake and PEP, we investigated the efficacy of PEP following an “on‐demand” (2‐1‐1) PrEP regimen [36] (schematic in Figure 4a). In our simulations, viral exposure occurs 2 (panels B and F), 5 (panels C and G) or 7 (panels D and H) days after the last PrEP “on demand” dose. A two‐ or three‐drug PEP regimen is then initiated within 0–72 hours post virus exposure (x‐axis) and continued for either for 14 (panels B–E) or 28 days (panels F–I).

Reading Figures 4b–d and f–h left‐to‐right shows that if the last PrEP‐on‐demand dosing event was 7 days ago, the added benefit of earlier PrEP‐on‐demand on subsequent PEP efficacy had almost vanished, compare to Figures 4e and i (no preceding PrEP). However, if PrEP‐on‐demand was taken less than 7 days prior to virus exposure, it increases subsequent PEP efficacy, as residual FTC‐TP and TFV‐DP concentrations may be present that either prevent acquisition in some individuals, delay sero‐conversion [37, 38] or result in a “pre‐loading” of drug concentrations for subsequent PEP. For example, if on‐demand‐PrEP was stopped 2 days prior to virus exposure, subsequent PEP with TDF/FTC may be *>*90% efficient, even when initiated within 3 days, Figure 4b,f. For the three‐drug PEP regimen, we observed a *>*90% efficacy even when on‐demand‐PrEP was stopped 5 days prior, provided that PEP was initiated within 48 hours after viral exposure and taken for *>*14 days, Figure 4c,g. Overall, we observe that past PrEP usage combined with PEP can increase efficacy.

Next, we investigated the concomitant impact of preceding daily PrEP with 1–7 average doses per week (denoted as 1/7–7/7), stopped 2 days before viral exposure, in conjunction with subsequent 2‐drug or 3‐drug PEP, initiated 2, 3 or 5 days after virus exposure and taken for 28 days (schematic in Figure 5a). As controls, we performed simulations without earlier PrEP (grey‐shaded areas), as well as PrEP‐only simulations (empty boxplots) in Figures 5b–d. Our simulations confirm the combined action of PrEP and PEP: Earlier PrEP boosts the efficacy of PEP, if PEP is initiated 2 or 3 days post‐exposure, Figure 5b,c. Compared both to “no‐PrEP” (grey‐shaded areas), as well as “no‐PEP” (empty boxplots), prophylactic efficacy is increased for the PrEP+PEP combination. However, PEP does not offer any additional protection when initiated 5 days post‐exposure (compare empty vs. coloured boxplots in Figure 5d). Interestingly, our model predicts that PrEP‐only with 100% adherence offers > 90% protection, when stopped 2 days before virus exposure (empty bars in Figure 5d). Also, for the PrEP+PEP combination, we observe > 90% protection, if 4/7 doses of earlier PrEP were taken and 3‐drug PEP was initiated 3 days post‐exposure. For comparison, PEP‐only offers only 50% (TDF/FTC+DTG) and 65% (TDF/FTC+EFV) protection if initiated 3 days post‐exposure (Figures 2c and 5c). If PEP is initiated 2 days post‐exposure, preceding PrEP may lift prophylactic efficacy from 90% (TDF/FTC+DTG) and 95% (TDF/FTC+EFV) to almost complete protection, if at least three‐out‐of‐seven versus two‐out‐of‐seven PrEP doses were taken and succeeding PEP contained TDF/FTC+DTG versus TDF/FTC+EFV.

Lastly, we tested scenarios in which the probability of PEP adherence declined substantially over time. We modelled PrEP with incomplete adherence 48 hours prior to virus exposure (schematic: Figure S4A). For exploratory purposes, we further assumed a substantial decrease in PEP adherence after 7 days, Figure S4B. Overall, compared to a full 28 days PEP regimen simulated in Figure 5b, we can see a drug‐specific decline in efficacy that is clearly seen in simulations without preceding PrEP (grey shaded area in Figure 5c): Two‐drug TDF/FTC is already quite inefficient (< 20%) when initiated 2, 3 or 5 days post‐exposure and hence poor PEP adherence has only a minimal further impact on its already low efficacy. (grey‐shaded areas in Figures 5b–d vs. Figures S4C–E). In contrast, for the three‐drug combinations, we see that poor PEP adherence negatively impacts on prophylactic efficacy (compare shaded areas in Figure 5b,c with Figure S4C,D). However, if ≥4/7 doses of earlier PrEP were taken and subsequent 3‐drug PEP was initiated ≤ 3 days post‐exposure, we predicted that prophylactic efficacy may exceed 90%.

In summary, we observe that preceding PrEP can substantially boost subsequent PEP efficacy for all drug regimens, if stopped 2 days before suspected virus exposure (Figure 4), or taken at 4/7 days on average (Figures 5 and S4). Moreover, preceding PrEP can “buy time” by slowing initial viral growth before PEP is initiated (compare schematic in Figure 1). Our simulations further highlighted that daily PrEP‐only with 100% adherence may provide > 90% protection, if stopped no more than 48 hours before exposure (Figures 5d and S4E). If PrEP was stopped 72 hours before exposure, prophylactic efficacy is 10% lower, compared to 48 hours, Figures S3 and S5.

## 4 DISCUSSION

The aim of this study was to evaluate the impact of delays in “time to PEP,” PEP duration and PrEP‐to‐PEP transition, based on a combined model of drug‐specific PK and viral dynamics. Our modelling by and large confirms UK, US and WHO guidelines on PEP [6, 7, 8], which recommend to combine a TDF/FTC backbone with a third drug, initiate PEP as early as possible and to take it for 28 days. Moreover, our simulations indicate that early PEP initiation after suspected virus exposure denotes the most critical parameter. For TDF/FTC two‐drug PEP, instantaneous (within 1 hour post‐exposure) initiation would be required. Adding a third drug to the TDF/FTC backbone “buys time.” However, protection may still be incomplete (Figure 2c), if a three‐drug PEP was initiated 72 hours post virus exposure and taken for 28 days. The duration of PEP is important to ensure that all replication‐competent virus is cleared (compare Figure 1a). EFV has a long half‐life compared to DTG (40–55 hours vs. 13.5–15.9 hours) [12, 13], such that therapeutic levels may persist for EFV, even after PEP is stopped. For EFV, the long half‐life may, therefore, increase the likelihood that the virus is cleared before the drug is washed out of the body (compare Figure 1a), making the duration of PEP intake a less sensitive parameter for EFV compared to DTG. While early PEP initiation may be particularly difficult in settings with less established health infrastructure, we found that individuals taking PrEP up to the time of exposure (−3 days) could re‐initiate the regimen and may add a third drug when it becomes available. The combined effects of PrEP+PEP in this scenario indicate synergy, which could arise from the fact that previous PrEP delays initial viral replication [38], or pre‐loads drug levels for subsequent PEP.

Our work has a number of limitations: Foremost, there is a lack of data that could be inputted into the model, due to a lack of clinical research into PEP. To strengthen the model, further clinical trials with clinically relevant endpoints may be required.

Our simulations refer to exposures with “wild type” viruses after typical sexual intercourse [16]. Notably, increasing inoculum sizes have a diminishing effect on prophylactic efficacy [30]. Moreover, non‐nucleoside reverse transcriptase inhibitor (NNRTI) drug resistance, which may amount to 10–20% of transmitted viruses in Africa and the Americas [39, 40] may severely diminish EFV‐based PEP efficacy [13] and thus the suitability of EFV as a PEP component. Notably, while we include EFV in our analysis to explore the impact of third‐drug components with very high molecular potency [41], we are not advocating EFV for PEP as it is contraindicated both for psychological side effects and low risk of serious liver toxicity. However, while some clinical trials suggest the superiority of integrase inhibitors (DTG over EFV) [42, 43, 44, 45] with regard to “time to viral load suppression,” we would like to emphasize that viral load kinetics decay more strongly for integrase inhibitors, merely because they inhibit a later stage of the viral replication cycle and not because of superior efficacy (or potency) [46, 47, 48]. Hence, the current preference for integrase inhibitors in PEP regimen should be motivated by tolerability and low prevalence of drug resistance rather than alleged efficacy. We did not investigate ritonavir‐boosted protease inhibitors lopinavir (LPV/r) or atazanavir (ATV/r) as third‐drug components in our model [7]. While these compounds have high molecular potency [41], we expect PEP efficacy to be similar to EFV. However, previous work suggests very steep dose‐response curves for LPV/r and ATV/r, implying that the prophylactic effect may rapidly drop in case of incomplete PEP adherence, or discontinuation [49]. In our model, we assume that the effect of the considered drugs is associated with systemic drug levels. Both EFV and DTG are lipophilic drugs that can rapidly cross cellular membranes by passive diffusion, such that their unbound drug concentration in plasma strongly correlates with effect‐site concentrations (“free drug hypothesis” [50, 51]) With regard to TDF/FTC, their phosphate moieties (TFV‐DP/FTC‐TP) in PBMCs were used as an effect marker, since our recent work [11] indicated strong correlation with effect, whereas concentrations in tissue homogenates were not predictive.

With regard to PD, we simulated synergistic effects between TFV‐DP and FTC‐TP, based on recent results [14] and assumed that the direct antiviral effects of DTG and EFV are additive to the TDF/FTC backbone, because either there was no evidence for non‐additivity (DTG) or parameters were lacking (EFV).

In our simulations, we modelled viral challenges after sexual exposure (receptive vaginal intercourse). Notably, the majority of non‐occupational PEP is administered after potential sexual exposure (PEPSE) [52] and women denote the major HIV risk group [53]. Occupational virus exposures, through for example needle‐stick injuries may lead to the translocation of larger amounts of viruses, which may negatively impact on prophylactic efficacy [30]. Consequently, the validity of our predictions with regard to occupational exposures warrants further ongoing investigation.

## 5 CONCLUSIONS

Our modelling suggests that “time to PEP” denotes the most critical parameter. Three‐drug PEP, preferably initiated no later than 48 hours after virus exposure, and taken for 28 days remains the optimal regimen. Three‐drug PEP for 14 days is less efficient than 28 days and 2‐drug (TDF/FTC) PEP only has high efficacy, if started within 1 hour after exposure. Self‐start 2‐drug (TDF/FTC) PEP with a subsequent addition of a third drug in the clinic works better than not self‐starting. Lastly, previous PrEP intake < 7 days prior to virus exposure boosts subsequent PEP efficacy and may widen the window period for “time to PEP” past 72 hours.

## COMPETING INTERESTS

JF received research funding from GSK for a shingles vaccine study. The remaining authors declare no competing interests.

## AUTHORS’ CONTRIBUTIONS

LZ and MvK wrote the manuscript with help from JF and SC. LZ and MvK designed the research. LZ performed the research, and LZ, MvK, JF and SC analysed the data.

## Supporting information




**Text S1**: This supplementary text contains the detailed viral dynamics model and the numerical approach (PGS) to compute the extinction probability.

**Figure S1**. Illustration of the viral dynamic model and the interference mechanisms of different drug classes.

**Figure S2**. Efficacy of TDF/FTC‐based PEP with delayed initiated double‐dose TDF/FTC and further delay of the third drug.

**Figure S3**. Predicted efficacy of once‐daily PEP, in case where PrEP was stopped 72hours before exposure.

**Figure S4**. Predicted efficacy of PEP with strongly declining adherence, in cases where PrEP was stopped 48hours before exposure.

**Figure S5**. Predicted efficacy of PEP with strongly declining adherence, in cases where PrEP was stopped 72hours before exposure.

## Data Availability

All data and computational codes are available at https://github.com/KleistLab/PEP [29].
