# Supplementary material for: Modelling the impact of initiation delay, duration and prior PrEP on the efficacy of post‐exposure prophylaxis containing a tenofovir/emtricitabine backbone
Source: J Int AIDS Soc. 2025 Jun 26;28(Suppl 1):e26454. doi: 10.1002/jia2.26454 (PMC12231637; doi:10.1002/jia2.26454)
Supplement: Supplementary file 1 — Text S1: This supplementary text contains the detailed viral dynamics model and the numerical approach (PGS) to compute the extinction probability. [file JIA2-28-e26454-s001.pdf]

Supplementary Text S1

Viral dynamics of HIV

We employ a viral dynamic model of HIV [1, 2], which contains six viral compartments: free infectious viruses V, early infected cells, i.e. T-cells T_1 and macrophages M_1, productively infected cells T_2 and M_2, and the latently infected T-cells T_L. As depicted in Fig S1, the dynamics can be defined by 15 reactions whose reaction propensities are denoted as a_1 through a_{15}. The drug classes that are investigated in this work are also integrated in this viral dynamics. Equations (S1)–(S15) define the concrete propensity of reaction.

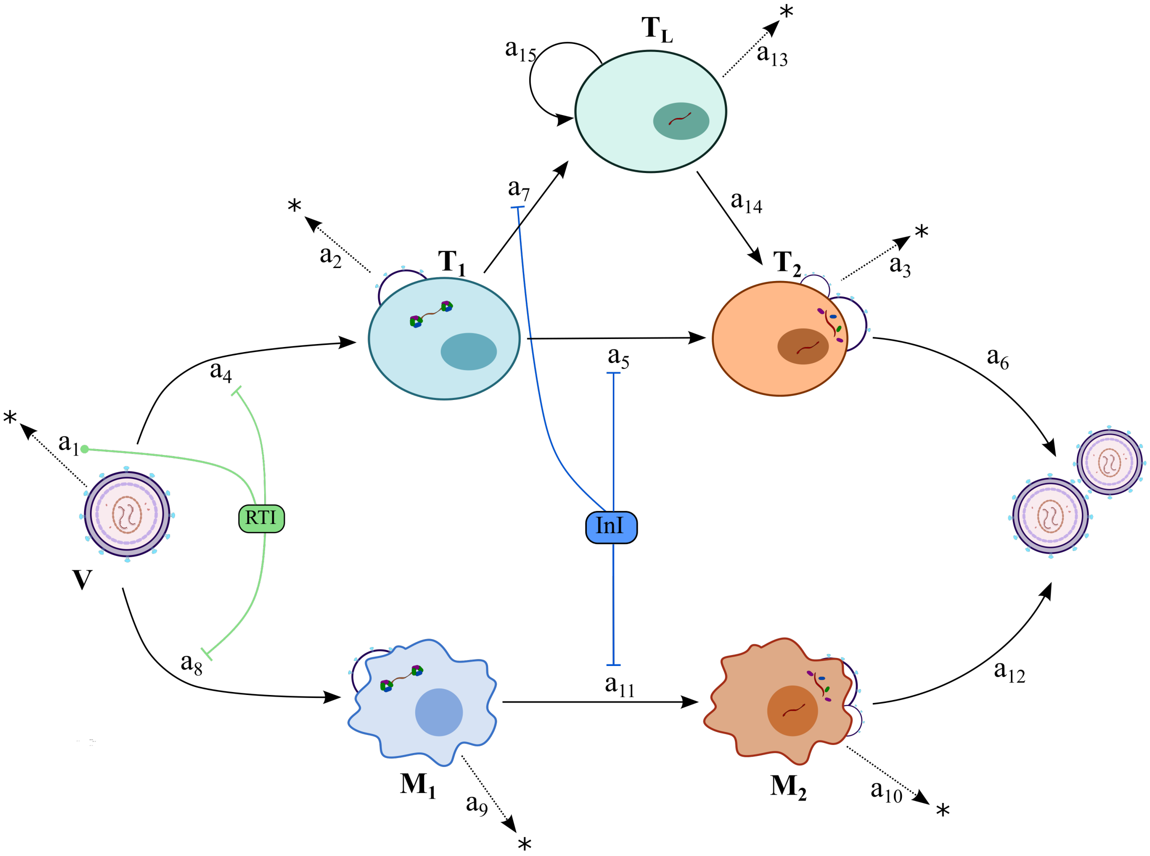

Figure S1. Illustration of the viral dynamic model and the interference mechanisms of different drug classes. Free infectious viruses V can infect target cells and create early infected T-cells T_1 and macrophages M_1 after successful infection. In early infected cells, the viral DNA can become integrated into the host genome, creating late infected cells T_2 and M_2, which are able to release new viruses. Early infected T-cells T_1 can become latently infected, i.e. the cells will transition into a resting state, denoted as T_L. The latent infected T-cells can replicate itself or be reactivated and turn into T_2 cells. Viral compartments can also be eliminated. $a_{j \in 1 \ldots 15}$ denotes the propensity of each reaction (see equations (S1)-(S15)). RTI: reverse transcriptase inhibitor; InI: integrase inhibitor.

R_1 : Clearance of free virus	$\mathrm{V} \to *$	$a_1(t) = (CL + (1+\eta(t)) \cdot (\beta_\mathrm{T} \cdot T_u + \beta_\mathrm{M} \cdot M_u)) \cdot V$	(S1)
R_2 : Clearance of T_1-cell	$\mathrm{T}_1 \to *$	$a_2 = (\delta_{\mathrm{PIC,T}} + \delta_{\mathrm{T}_1}) \cdot T_1$	(S2)
R_3 : Clearance of T_2-cell	$\mathrm{T}_2 \to *$	$a_3 = \delta_{\mathrm{T}_2} \cdot T_2$	(S3)
R_4 : Infection of susceptible T-cell	$\mathrm{V} \to \mathrm{T}_1$	$a_4(t) = (1-\eta(t)) \cdot \beta_\mathrm{T} \cdot T_u \cdot V$	(S4)
R_5 : Integration of viral DNA into T_1	$\mathrm{T}_1 \to \mathrm{T}_2$	$a_5(t) = (1-\eta(t)) \cdot (1-p) \cdot k_\mathrm{T} \cdot T_1$	(S5)
R_6 : Production of new virus	$\mathrm{T}_2 \to \mathrm{V}+\mathrm{T}_2$	$a_6 = N_\mathrm{T} \cdot T_2$	(S6)
R_7 : Transition of T_1 into latent infection	$\mathrm{T}_1 \to \mathrm{T}_\mathrm{L}$	$a_7(t) = (1-\eta(t)) \cdot p \cdot k_\mathrm{T} \cdot \mathrm{T}_1$	(S7)
R_8 : Infection of susceptible macrophage	$\mathrm{V} \to \mathrm{M}_1$	$a_8(t) = (1-\eta(t)) \cdot \beta_M \cdot M_u \cdot V$	(S8)
R_9 : Clearance of M_1-cell	$\mathrm{M}_1 \to *$	$a_9 = (\delta_{\mathrm{PIC,M}} + \delta_{\mathrm{M}_1}) \cdot M_1$	(S9)
R_{10} : Clearance of M_2-cell	$\mathrm{M}_2 \to *$	$a_{10} = \delta_{\mathrm{M}_2} \cdot M_2$	(S10)
R_{11} : Integration of viral DNA into M_1	$\mathrm{M}_1 \to \mathrm{M}_2$	$a_{11}(t) = (1-\eta(t)) \cdot k_\mathrm{M} \cdot M_1$	(S11)
R_{12} : Production of new virus	$\mathrm{M}_2 \to \mathrm{V}+\mathrm{M}_2$	$a_{12} = N_\mathrm{M} \cdot M_2$	(S12)
R_{13} : Clearance of T_L-cell	$\mathrm{T}_\mathrm{L} \to *$	$a_{13} = \delta_L \cdot T_L$	(S13)
R_{14} : Reactivation of T_L-cell	$\mathrm{T}_\mathrm{L} \to \mathrm{T}_2$	$a_{14} = \alpha \cdot T_L$	(S14)
R_{15} : Replication of T_L-cell	$\mathrm{T}_\mathrm{L} \to \mathrm{T}_\mathrm{L}+\mathrm{T}_\mathrm{L}$	$a_{15} = \zeta \cdot T_L$	(S15)

Here we assume a continuous virus production model, where T_2 and M_2 cells will produce viruses continuously until they are eliminated. The used parameters except for the replication rate of T_L are listed in [2], Table 1 therein. The T_L replication rate ζ is adopted from [3].

Numerical approach for PEP efficacy

In the *Methods* section we defined the prophylactic efficacy of a given prophylactic regimen, as the reduction in infection probability *per exposure*. Since extinction probability is the complement of infection probability, we can compute the extinction probability by adopting a recently developed numerical approach [4]. Based on the viral dynamics model in Fig S1, the extinction probability P_E of each single viral compartment can be derived as follows:

$$
\begin{aligned}
\frac{dP_E(\hat{V})}{dt} &= (a_1(t) + a_4(t) + a_8(t)) \cdot P_E(\hat{V}) - a_4(t) \cdot P_E(\hat{T}_1) - a_8(t) \cdot P_E(\hat{M}_1) - a_1(t) \\
\frac{dP_E(\hat{T}_1)}{dt} &= (a_2 + a_5(t) + a_7(t)) \cdot P_E(\hat{T}_1) - a_5(t) \cdot P_E(\hat{T}_2) - a_7(t) \cdot P_E(\hat{T}_L) - a_2 \\
\frac{dP_E(\hat{T}_2)}{dt} &= (a_3 + a_6) P_E(\hat{T}_2) - a_6 \cdot P_E(\hat{V}) \cdot P_E(\hat{T}_2) - a_3 \\
\frac{dP_E(\hat{T}_L)}{dt} &= (a_{13} + a_{14} + a_{15}) \cdot P_E(\hat{T}_L) - a_{14} \cdot P_E(\hat{T}_2) - a_{15} \cdot P_E(\hat{T}_L)^2 - a_{13} \\
\frac{dP_E(\hat{M}_1)}{dt} &= (a_9 + a_{11}(t)) P_E(\hat{M}_1) - a_{11}(t) \cdot P_E(\hat{M}_2) - a_9 \\
\frac{dP_E(\hat{M}_2)}{dt} &= (a_{10} + a_{12}) P_E(\hat{M}_2) - a_{12} \cdot P_E(\hat{V}) \cdot P_E(\hat{M}_2) - a_{10}
\end{aligned}
\qquad \text{(S16)}
$$

The time-dependent reaction rates are given in eqs (S1)–(S15). The system of ordinary differential equations (S16) is solved backwards using standard ODE solvers, as outlined in [4].

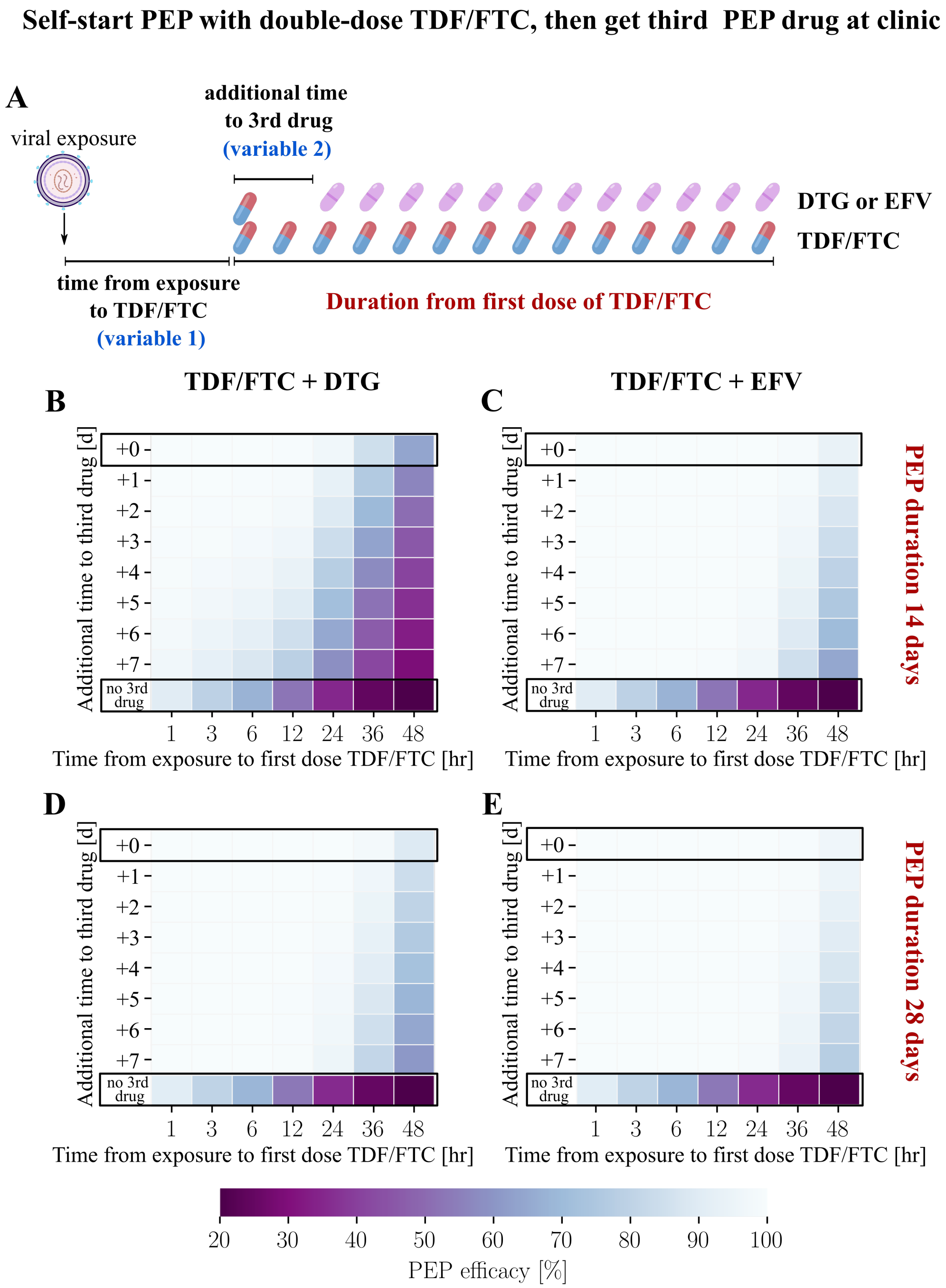

Figure S2. Efficacy of TDF/FTC-based PEP with delayed initiated double-dose TDF/FTC and further delay of the third drug. A:Schematic of the dosing regimen. For the drug combinations TDF/FTC + DTG and TDF/FTC + EFV, PEP efficacy was computed for virus exposures occurring within 1 to 48 hours before the first double dose of TDF/FTC. The third drug was then added to the PEP regimen 1 to 7 days after the the first dose of TDF/FTC. B: PEP efficacy for the drug combination TDF/FTC + DTG, PEP duration was 14 days from the first dose of TDF/FTC. C: Corresponding PEP efficacy for TDF/FTC + EFV. D: PEP efficacy for TDF/FTC + DTG when taken for 28 days after the first TDF/FTC dose. E: Corresponding PEP efficacy for TDF/FTC + EFV. The daily oral dose for each drug corresponds to 300/200mg TDF/FTC, 50mg DTG and 400mg EFV. In panel B-E, the top row outlined in black denotes the scenario where the third drug is immediately added to the TDF/FTC backbone; the bottom row represents the scenario where no third drug was added to the TFC/TDF backbone. PEP, post-exposure prophylaxis. TDF, tenofovir disoproxil fumarate. FTC, emtricitabine. DTG, dolutegravir. EFV, efavirenz

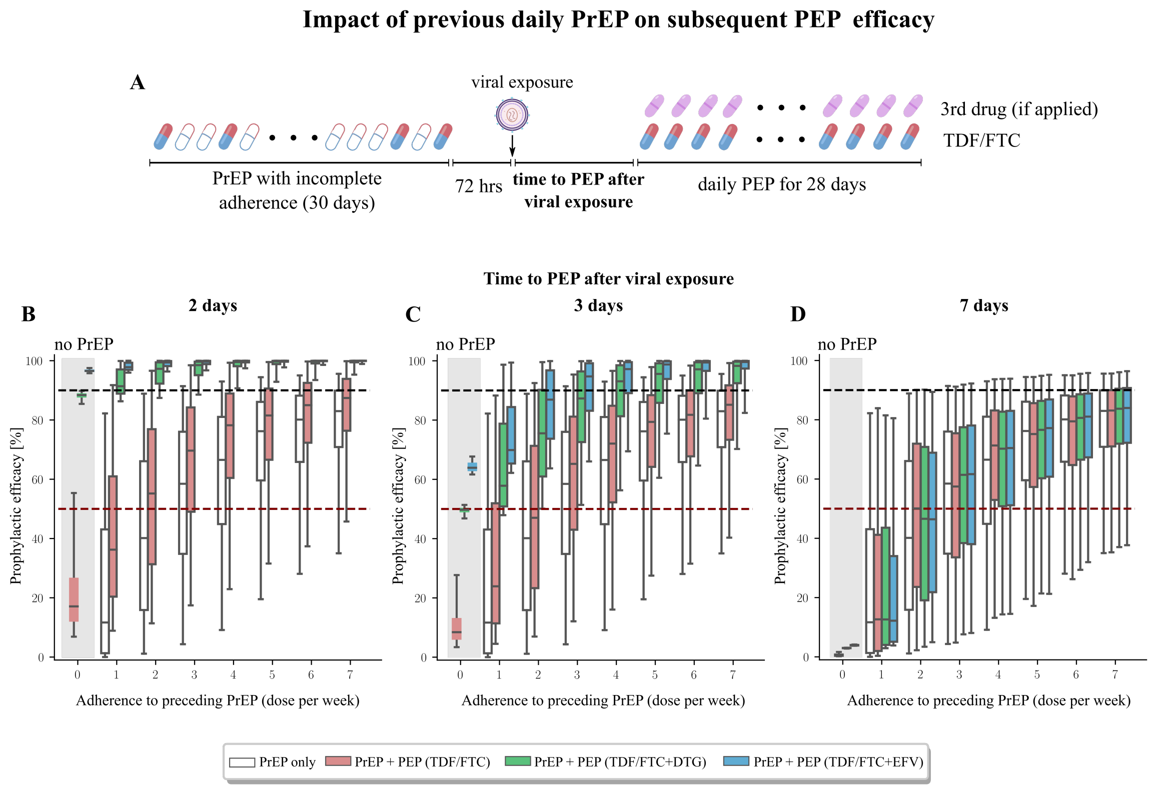

Figure S3. Predicted efficacy of once-daily PEP, in case where PrEP was stopped 72hours before exposure. A: Schematic of dosing regimen: PrEP with incomplete, variable levels of adherence was taken and stopped 72hours before virus exposure. PEP with either TDF/FTC, or TDF/FTC + DTG or EFV was then initiated after a variable delay and taken for 28 days. PEP efficacy is calculated with regards to preceding PrEP adherence, as well as delay in PEP initiation. B-D: Computed prophylactic efficacy for the distinct PrEP+PEP regimen, if PEP was initiated 2, 3, or 7 days post-exposure and taken daily for 28days. The daily oral dose for each drug corresponds to 300/200mg TDF/FTC, 50mg DTG and 400mg EFV. The grey-shaded area indicates PEP efficacy, with no prior PrEP, while empty boxplots highlight the prophylactic effect of preceding PrEP, without subsequent PEP. Boxplots show the median, interquartile ranges and whiskers encompass the 95% confidence interval. Dashed red lines indicate 50% prophylactic efficacy, while dashed black lines indicate 90% prophylactic efficacy. PEP, post-exposure prophylaxis. PrEP, pre-exposure prophyloaxis. TDF, tenofovir disoproxil fumarate. FTC, emtricitabine. DTG, dolutegravir. EFV, efavirenz

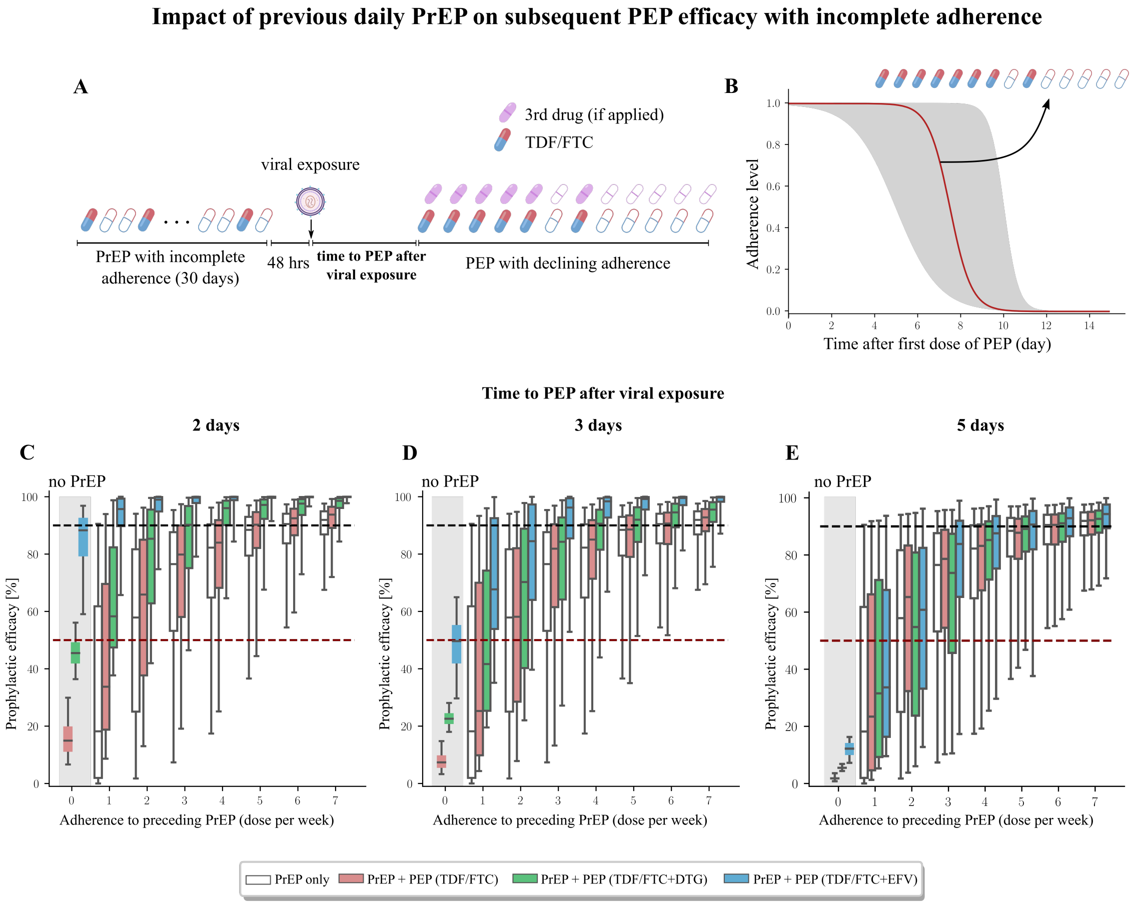

Figure S4. Predicted efficacy of PEP with strongly declining adherence, in cases where PrEP was stopped 48hours before exposure. A: Schematic of dosing regimen: PrEP with incomplete, variable levels of adherence was taken and stopped 48 hours before virus exposure. PEP with either TDF/FTC, or TDF/FTC + DTG or EFV was then initiated after a variable delay and adherence strongly decreases over time. PEP efficacy is calculated with regards to preceding PrEP adherence, as well as delay in PEP initiation. B: Simulated PEP adherence probability with a half maximum at 7 days post PEP initiation. C–E: Computed prophylactic efficacy for the distinct PrEP+PEP regimen, if PEP was initiated 2, 3, or 5 days post-exposure and adherence declined substantially after 7 days. The daily oral dose for each drug corresponds to 300/200mg TDF/FTC, 50mg DTG and 400mg EFV. The grey-shaded area indicates PEP efficacy without prior PrEP, while empty boxplots highlight the prophylactic effect of preceding PrEP, without subsequent PEP. Boxplots show the median, interquartile ranges and whiskers encompass the 95% confidence interval. Dashed red lines indicate 50% prophylactic efficacy, while dashed black lines indicate 90% prophylactic efficacy. PEP, post-exposure prophylaxis. PrEP, pre-exposure prophyloaxis. TDF, tenofovir disoproxil fumarate. FTC, emtricitabine. DTG, dolutegravir. EFV, efavirenz

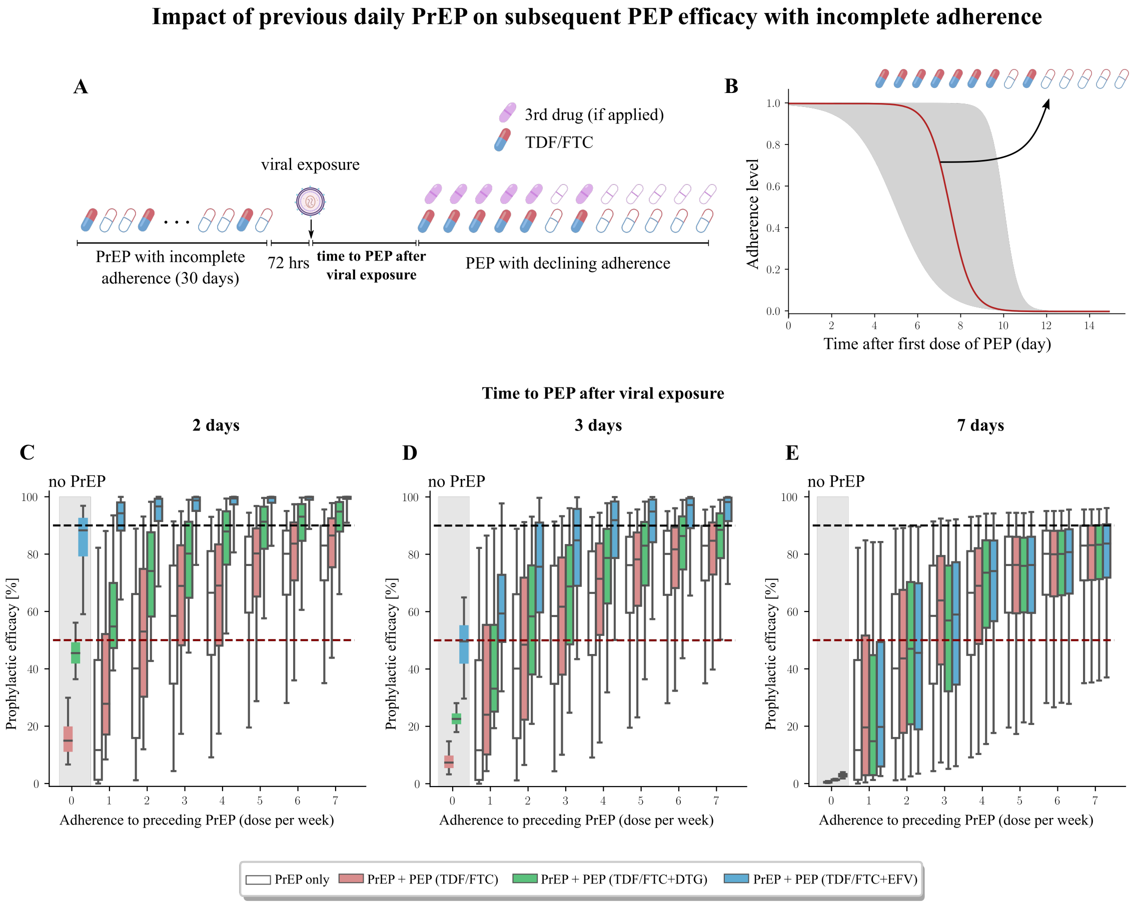

Figure S5. Predicted efficacy of PEP with strongly declining adherence, in cases where PrEP was stopped 72hours before exposure. A: Schematic of dosing regimen: PrEP with incomplete, variable levels of adherence was taken and stopped 72 hours before virus exposure. PEP with either TDF/FTC, or TDF/FTC + DTG or EFV was then initiated after a variable delay and adherence strongly decreases over time. PEP efficacy is calculated with regards to preceding PrEP adherence, as well as delay in PEP initiation. B: Simulated PEP adherence probability with a half maximum at 7 days post PEP initiation. C–E: Computed prophylactic efficacy for the distinct PrEP+PEP regimen, if PEP was initiated 2, 3, or 7 days post-exposure and adherence declined substantially after 7 days. The daily oral dose for each drug corresponds to 300/200mg TDF/FTC, 50mg DTG and 400mg EFV. The grey-shaded area indicates PEP efficacy without prior PrEP, while empty boxplots highlight the prophylactic effect of preceding PrEP, without subsequent PEP. Boxplots show the median, interquartile ranges and whiskers encompass the 95% confidence interval. Dashed red lines indicate 50% prophylactic efficacy, while dashed black lines indicate 90% prophylactic efficacy. PEP, post-exposure prophylaxis. PrEP, pre-exposure prophyloaxis. TDF, tenofovir disoproxil fumarate. FTC, emtricitabine. DTG, dolutegravir. EFV, efavirenz

References

1. Max Von Kleist, Stephan Menz, and Wilhelm Huisinga. Drug-class specific impact of antivirals on the reproductive capacity of HIV. *PLOS Computational Biology*, 6(3):e1000720, 2010.

2. Max von Kleist, Stephan Menz, Hartmut Stocker, Keikawus Arasteh, Christof Schütte, and Wilhelm Huisinga. HIV quasispecies dynamics during pro-active treatment switching: impact on multi-drug resistance and resistance archiving in latent reservoirs. *PlOS ONE*, 6(3):e18204, 2011.

3. Daniel B Reeves, Elizabeth R Duke, Thor A Wagner, Sarah E Palmer, Adam M Spivak, and Joshua T Schiffer. A majority of HIV persistence during antiretroviral therapy is due to infected cell proliferation. *Nature communications*, 9(1):1–16, 2018.

4. Lanxin Zhang, Junyu Wang, and Max von Kleist. Numerical approaches for the rapid analysis of prophylactic efficacy against HIV with arbitrary drug-dosing schemes. *PLOS Computational Biology*, 17(12):e1009295, 2021.
